# Mechanisims of asthma and allergic disease – 1094. Pre-clinical characterization of RP3133, a novel and potent CRAC channel inhibitor for the treatment of respiratory disorders

**DOI:** 10.1186/1939-4551-6-S1-P90

**Published:** 2013-04-23

**Authors:** Kasi Viswanath Routhu, Sridhar Veeraraghavan, Gayatri Swaroop Merikapudi, Babu G, Srikant Viswanadha, Swaroop Vakkalanka

**Affiliations:** 1 Pharmacology, Incozen Therapeutics Pvt. Ltd., Hyderabad, India

## Introduction

Calcium release activated calcium channels inhibitors have a potent role in treatment of autoimmune disorders mediated dysregulated T-lymphocyte and mast cell functioning. Herein, we describe the pre-clinical of RP3133, a novel and potent CRAC channel inhibitor with scope for development as a clinical candidate for asthma.

## Methods

Inhibition of CRAC channel activity in Jurkat cells, cytokine release from human whole blood or PBMC, and mast cell degranulation were estimated. *In vivo* efficacy of the compound was determined in experimental models of asthma in guinea pigs including PAF or ovalbumin induced eosinophil infiltration into lungs ovalbumin induced histamine release from mast cells, and airway hyper-responsiveness.

## Results

RP3133 significantly inhibited calcium entry into Jurkat cells (**38 nM**) besides reducing IL-4 **(<550 nM**) and IL-5 **(<750 nM**) release from human whole blood and PBMC. Additionally, the compound suppressed IgE-induced mast cell degranulation at nanomolar concentrations (**139 nM**). Oral administration of RP3133 in guinea pigs resulted in a dramatic reduction in eosinophil infiltration in an acute model of PAF-induced allergic asthma (**ED_50_ = 0.2 mg/kg/p.o**) as well as in an experimental model of ovalbumin-induced chronic airway inflammation (**ED_50_ = 2.5 mg/kg/p.o)**. Additionally, RP3133 caused a significant reduction (***P*<*0.05***) citric acid, histamine, or methacholine induced airway resistance in sensitized guinea pigs. Consistent with *in vitro* findings, the compound caused a significant inhibition of mast cell degranulation manifested by a reduction in histamine release.

## Conclusions

Results demonstrate the potential of RP3133 as an anti-asthmatic agent as evidenced from pre-clinical data. Toxicological evaluation of the molecule is currently in progress.

